# Association and *cis*-mQTL analysis of variants in serotonergic genes associated with nicotine dependence in Chinese Han smokers

**DOI:** 10.1038/s41398-018-0290-8

**Published:** 2018-11-07

**Authors:** Haijun Han, Qiang Liu, Zhongli Yang, Mu Wang, Yunlong Ma, Liyu Cao, Wenyan Cui, Wenji Yuan, Thomas J. Payne, Lanjuan Li, Ming D. Li

**Affiliations:** 1 State Key Laboratory for Diagnosis and Treatment of Infectious Diseases, The First Affiliated Hospital, Collaborative Innovation Center for Diagnosis and Treatment of Infectious Diseases, Zhejiang University School of Medicine, Hangzhou, China; 2 ACT Center for Tobacco Treatment, Education and Research, Department of Otolaryngology and Communicative Sciences, University of Mississippi Medical Center, Jackson, MS USA; 3 Research Center for Air Pollution and Health, Zhejiang University, Hangzhou, China; 4 Institute of Neuroimmune Pharmacology, Seton Hall University, South Orange, NJ USA

## Abstract

Variants in serotonergic genes are implicated in nicotine dependence (ND) in subjects of European and African origin, but their involvement with smoking in Asians is largely unknown. Moreover, mechanisms underlying the ND risk-associated single-nucleotide polymorphisms (SNPs) in these genes are rarely investigated. The Fagerström Test for Nicotine Dependence (FTND) score was used to assess ND in 2616 male Chinese Han smokers. Both association and interaction analysis were used to examine the association of variants in the serotonergic genes with FTND. Further, expression and methylation quantitative trait loci (*cis*-mQTL) analysis was employed to determine the association of individual SNPs with the extent of methylation of each CpG locus. Individual SNP-based association analysis revealed that rs1176744 in *HTR3B* was marginally associated with FTND (*p* = 0.042). Haplotype-based association analysis found that one major haplotype, T-T-A-G, formed by SNPs rs3758987-rs4938056-rs1176744-rs2276305, located in the 5′ region of *HTR3B*, showed a significant association with FTND (*p* = 0.00025). Further, a significant genetic interactive effect affecting ND was detected among SNPs rs10160548 in *HTR3A*, and rs3758987, rs2276305, and rs1672717 in *HTR3B* (*p* = 0.0074). Finally, we found four CpG sites (CpG_4543549, CpG_4543464, CpG_4543682, and CpG_4546888) to be significantly associated with three *cis*-mQTL SNPs (i.e., rs3758987, rs4938056, and rs1176744) located in our detected haplotype within *HTR3B*. In sum, we showed SNP rs1176744 (Tyr129Ser) to be associated with ND. Together with the SNPs rs3758987 and rs4938056 in *HTR3B*, they formed a major haplotype, which had significant association with ND. We further showed these SNPs contribute to ND through four methylated sites in *HTR3B*. All these findings suggest that variants in the serotonergic system play an important role in ND in the Chinese Han population. More importantly, these findings demonstrated that the involvement of this system in ND is through gene-by-gene interaction and methylation.

## Introduction

Cigarette smoking is still a serious issue all over the world. Each year, smoking causes about six million deaths worldwide, of which more than five million result directly from cigarette smoking^1^. Although smoking prevalence has decreased greatly since 1990^2^, the annual number of smoking-related deaths in the world is projected to rise to more than eight million by 2030^3,4^. In China, cigarette consumption was approximately twofold higher in 2016 than it was in 1998^5^, and the prevalence of smoking differs greatly in men and women in different regions^6,7^. A recent report showed that there is a high smoking prevalence in Chinese men, and most of these users are highly nicotine-dependent (ND)^8^.

Cigarette smoking is a complex behavior that is influenced by both environmental and genetic factors^9^. Nicotine is one of the primary ingredients of cigarette smoke and is addictive. Meta-analysis of twin and family studies has found the average heritability of ND to be 0.59 in males and 0.46 in females with an average heritability of 0.56 in both males and females^10^. Nicotine exerts its biological effects by binding to nicotinic acetylcholine receptors (nAChRs) located in various brain regions^11^, where it either stimulates or inactivates them through desensitization^12^. The activation of nAChRs in the striatum stimulates dopamine release after nicotine exposure^13,14^. Nicotine can also induce the release of various other neurotransmitters in the brain, such as catecholamines, serotonin (5-hydroxytryptamine, 5-HT), and peptides^15^.

5-HT is a polyfunctional signaling molecule that acts mainly as a neurotransmitter within both the central and peripheral nervous systems^16,17^. The diverse effects of 5-HT are mediated by 5-HT receptors, which are grouped into seven classes (5-HT1 through 5-HT7)^18^. Most of these are G protein-coupled receptors, but the 5-HT3 receptors belong to the Cys-loop superfamily of pentameric neurotransmitter-gated ion channels^19,20^. The 5-HT3 receptors share similarities with acetylcholine, subtype A of γ-aminobutyric acid and glycine receptors, with about 30% structural and functional homology^21^. Both 5-HT3 receptors and nAChRs exist widely in the central nervous system but are localized to presynaptic and postsynaptic sites^22,23^. Also, nAChRs are co-localized with 5-HT3 serotonin receptors on the same presynaptic striatal nerve terminals regulating dopamine neurotransmission, indicating a convergence of cholinergic and serotonergic systems in the striatum^24^.

To date, five 5-HT3 receptor subunit genes (*HTR3A*, *HTR3B*, *HTR3C*, *HTR3D*, and *HTR3E*) have been identified in humans that encode five receptor subunits, 5-HT3_A_, 5-HT3_B_, 5-HT3_C_, 5-HT3_D_, and 5-HT3_E_^19^. Of these, the 5-HT3_A_ and 5-HT3_B_ subunits are more commonly investigated. Both human *HTR3A* and *HTR3B* are located within a 90-kb region on chromosome 11q23.1^25^. *SLC6A4* or *5-HTT* is an important serotonin transporter gene, which is located on chromosome 17q11.2 and encodes a membrane protein that regulates synaptic 5-HT concentrations through recycling 5-HT from the synaptic cleft^26^.

Serotonergic genes have been extensively investigated in various conditions, such as psychiatric^27,28^ and eating^29^ disorders. As a result of 5-HT3 receptor activation, the serotonin-gated ion channel undergoes rapid depolarization and desensitization, then releases stored neurotransmitter, suggesting an important role of the serotonergic system in neuronal circuitry involved in drug abuse^30^. The serotonergic system was reported to be related to the effects of nicotine, leading to the development of ND^31^. Recently, there were some genetic studies on serotonergic genes in drug abuse, for instance, smoking^32^, alcohol dependence^33–35^, and other drugs dependencies^33,36^. By using various genetic approaches such as genome-wide linkage, candidate gene association, and genome-wide association analysis, this research has provided insights into the genetic architecture of ND^37^. However, there have been limited genetic studies on the relation of serotonergic genes to smoking in Chinese populations.

Thus, in this study, we applied various genetic analyses to investigate the role of serotonergic genes in ND in a less commonly investigated population, Chinese Han smokers. We first performed association analysis at both the individual single-nucleotide polymorphism (SNP) and haplotype levels. We then determined interactive effects among serotonergic genes to understand their roles in ND. Finally, to explore the underlying mechanisms of ND, we established a link for those risk variants for ND with differential DNA methylation loci by performing methylation quantitative trait locus (*cis*-mQTL) analysis.

## Materials and methods

### Samples

The donors of the samples used in the present study were enrolled from local hospitals in Jincheng and Taiyuan in Shanxi Provinces, China, during 2012–2013^8^. Because only about 2% of Chinese women smoke^38^, only male smokers (*N* = 2616) were included in the study. For each subject, detailed information on age, education, income, medical history, environment, and smoking-related behaviors were collected by trained interviewers. For this study, we used the Fagerström Test for Nicotine Dependence (FTND) score (0–10 scale), one of the most commonly used instruments to assess smoking quantity (SQ)^39,40^, to assess the ND of each smoker. On the basis of previous reports, smokers with an FTND score ≥ 6 were considered to be highly ND, and those with an FTND of <6 were classified as moderately or slightly dependent^40,41^. Supplementary Table 1 shows the distribution of FTND scores of smokers in our sample, which included 1243 heavy and 1373 light smokers. All subjects provided written informed consent for participating in the study, which was approved by the Institutional Review Board of the First Affiliated Hospital of Zhejiang University School of Medicine.

We used Structure 2.3.4^42^ to assess population stratification according to the genotyping data of a group of 30 ancestry-informative marker panel. The parameters of simulation were set as 100 000 burns-in and 100 000 iterations and the *K* to 2 because the samples were collected from two areas. We pooled those two groups for further analysis, as no population admixture was revealed (Supplementary Figure 1).

### SNP selection

According to the minor allele frequency (MAF) > 0.05 based on the National Center for Biotechnology Information (NCBI) dbSNP database (http://www.ncbi.nlm.nih.gov/SNP/) and published papers^32,36,43^, 8 SNPs in *HTR3A*, 8 SNPs in *HTR3B*, and 1 SNP in *SLC6A4* were selected for genotyping. Detailed information about each SNP is shown in Supplementary Table 2. To ensure reliable association results, we selected SNPs according to the following criteria: genotype calling rate > 95%, MAF > 0.01, and Hardy-Weinberg equilibrium (HWE) *p* > 0.05. Under these criteria, SNPs rs1150226 and rs33940208 in *HTR3A*, and rs11606194, rs17614942, and rs3782025 in *HTR3B* were excluded from the analysis because of their low genotyping quality. After removing these SNPs, 12 SNPs were available for subsequent association analysis.

### DNA sample processing and genotyping

For each participant, peripheral blood was collected, and the genomic DNA was extracted using the Gentra Puregene blood kit according to the manufacturer’s protocol. The DNA concentration and optical density were measured using NanoDrop 2000, and a *A*_260_/*A*_280_ ratio of 1.7–1.9 was required prior to genotyping. The starting normalized DNA concentration of each sample was set at 50 ng/μl. The QuantStudio™ OpenArray^®^ AccuFill™ System (Applied Biosystems, Inc.) was used to transfer samples and load the OpenArray^®^ plate. After sealing of the plate, the OpenArray^®^ case was loaded with OpenArray^®^ Immersion Fluid. Finally, the OpenArray^®^ Plug was inserted into the port, and the plug was twisted clockwise until hand‐tight, when it was ready to be loaded into the QuantStudio™ 12K Flex System (Applied Biosystems). The DNA genotyping was performed using the QuantStudio™ 12K Flex OpenArray platform, which is based on the *Taq*Man genotyping assay where two probes were labeled with FAM or VIC fluorescent dye. After 50 cycles of amplification, the fluorescent signals were recorded. Further analysis was performed with Life Technologies *Taq*Man Genotyper (v. 1.3) software. All SNPs were called using an “autocall” feature; further, a manual inspection of the autocalled data was performed by two independent researchers. As noted, samples with a call rate of <95% were removed from subsequent association analysis. The threshold for the genotyping rate of all SNPs was set as ≥95%, and the *p* value for the HWE was set as no ≥5%. All procedures and conditions used in this study were essentially the same as what we reported previously on the same samples^44,45^.

### DNA methylation dataset

The DNA methylation dataset used here consisted of samples from 36 smokers and 36 nonsmokers selected from the sample set described above on the basis of the age, sex, and smoking status, which is an ongoing whole-genome bisulfite sequencing project in this laboratory^45^. Briefly, the Illumina HiSeq X Ten platform was applied to conduct DNA methylation sequencing with an average of about 700 million (±75 million; SD) 150-bp paired-end reads per sample. Clean reads were mapped to the hg19 reference genome using Bismark^46^. Then, we combined two strands of information of CpG sites and excluded CpGs with reads <5 or those overlapping with common variants in the Chinese Han genome (MAF > 0.05).

### Statistical analysis

#### Individual-based association analysis

For all 12 individual SNPs in *HTR3A*, *HTR3B*, and *SLC6A4*, association analysis with the FTND score was performed using a logistic regression model with the PLINK program (v. 1.07)^47^. The additive genetic model was employed for all analyses with age, working time, and area as covariates. Because the samples were collected from coal miners, and because of the different jobs held by the various subjects, some of them needed to work underground for more than 8 h/day, whereas others, such as technical persons, worked primarily aboveground. It is thus necessary to consider “working time” as a covariate in the analysis. In addition, the samples were collected from two areas of Shanxi province (i.e., Taiyuan and Jincheng), so area was treated as another covariate. A *p* value of <0.05 was considered statistically significant after correction for multiple testing using the Bonferroni procedure.

#### Haplotype-based association analysis

Only major haplotypes with a frequency of >5% were included in the analysis. Haploview software (v. 4.2) was used to determine pair-wise linkage disequilibrium and haplotype blocks^48,49^. Association analysis with the FTND score was performed using Haplo Stats (v. 1.7.7)^50^. For the approach used for performing haplotype-based association analysis, please refer to our previous reports^43,44^.

#### Gene–gene interaction analysis

Genetic interactive effects of *HTR3A*, *HTR3B*, and *SLC6A4* were analyzed using the GPU-based Generalized Multifactor Dimensionality Reduction (GMDR-GPU) program developed by our research team^51^. An exhaustive search was performed for all possible 2- to 5-locus combination models. The interactive effects among the 12 SNPs were calculated with FTND score according to a generalized linear model by including age, working time, and area as covariates. Three criteria were used to determine the best gene–gene interaction model: (1) the cross-validation consistency (CVC) statistics for the selected variant combinations; (2) the prediction accuracy; and (3) significant *p* value, which was determined by 10^7^ permutation tests for the selected SNP combinations. The CVC is the number of times the particular combination is selected from all the training sets. The higher the CVC, the more robust the SNP combination as a predictive interaction model. After identifying the candidate interaction models, their prediction accuracies are calculated by averaging their corresponding testing accuracies among all the data partitions that are not contained in the training sets. The significance or *p* value is calculated by a permutation test based on the prediction accuracy. Based on 10^7^ permutation tests, CVC > 7 of 10, prediction accuracy >55%, and empirical *p* value < 0.005 are considered a significant SNP combination.

#### *cis*-mQTL analysis

We conducted *cis*-mQTL analysis with a similar procedure, as described before^45^. The region for the *cis-*mQTL analysis was set within 50 kb upstream and downstream of each SNP of interest^52,53^. Prior to *cis*-mQTL analysis, we removed the low-quality DNA methylation sites, those with a calling rate < 80%. Four SNPs (rs3758987, rs4938056, rs1176744, and rs2276305) formed into a haplotype in *HTR3B* were chosen for the *cis*-mQTL association analysis. The Matrix eQTL (v. 2.1.1) R package^54^ was used to test association of the four risk SNPs with the extent of methylation of each CpG site using a linear regression model.

Because there were both smokers and nonsmokers in the cohort yielding the methylation data, we adjusted smoking status and age as covariates to detect the effects of each risk variant with adjacent extents of methylation. Significant associations were defined after Bonferroni correction (*p* < 0.05/4788 = 1.04 × 10^−5^), where 4788 is the total number of association tests for the four SNPs (i.e., 1175 CpG sites for rs1176744, 1173 CpG sites for rs2276305, 1221 CpG sites for rs3758987, and 1219 CpG sites for rs4938056). In addition, we performed a *cis*-mQTL analysis within smokers using the same method Table 1.

**Table 1:** Characteristics of samples used in the study

Characteristic	Number (total/mean)	SD	Range
No. of subjects	2,616	NA	NA
Age (years)	40.4	9.7	19–74
FTND score	5.45	1.41	0–10

*FTND* Fagerström Test for Nicotine Dependence, *SD* standard deviation, *NA* not available

## Results

### Individual SNP-based association analysis

The results from the individual SNP-based association analyses with FTND score are shown in Table 2. Of the 12 SNPs, rs1176744 in *HTR3B* is the only one showing a significant association with FTND score under the additive model (*β* = −0.10; *p* = 0.042). However, it was no longer significant after Bonferroni correction for multiple testing. We found no significance of the SNPs in *HTR3A* and *SLC6A4* (*p* > 0.05).

**Table 2:** Individual SNP-based association analysis with FTND in Chinese Han smokers

Gene	SNP ID	MAF	Statistics^a^	
			Beta	*p* Value
*HTR3A*	rs2276302	G (0.10)	0.07	0.221
	rs1150220	A (0.09)	0.09	0.166
	rs1062613	T (0.09)	0.08	0.236
	rs1176713	G (0.24)	0.05	0.283
	rs1985242	A (0.28)	0.03	0.413
	rs10160548	G (0.34)	0.00	0.989
*HTR3B*	rs1176744	C (0.17)	−0.10	**0.042**
	rs3758987	C (0.17)	−0.08	0.098
	rs1672717	G (0.38)	−0.05	0.202
	rs2276305	A (0.27)	−0.04	0.294
	rs4938056	C (0.41)	−0.02	0.664
*SLC6A4*	rs1042173	A (0.20)	0.05	0.330

Age, working time, and area were used as covariates. *p* Values < 0.05 are shown in boldface type. The corrected *p* value after Bonferroni correction for 12 tested SNPs is 0.0042 (0.05/12)

*MAF* minor allele frequency

^a^Additive model was used in the analyses

### Haplotype-based association analysis

**Fig. 1:**
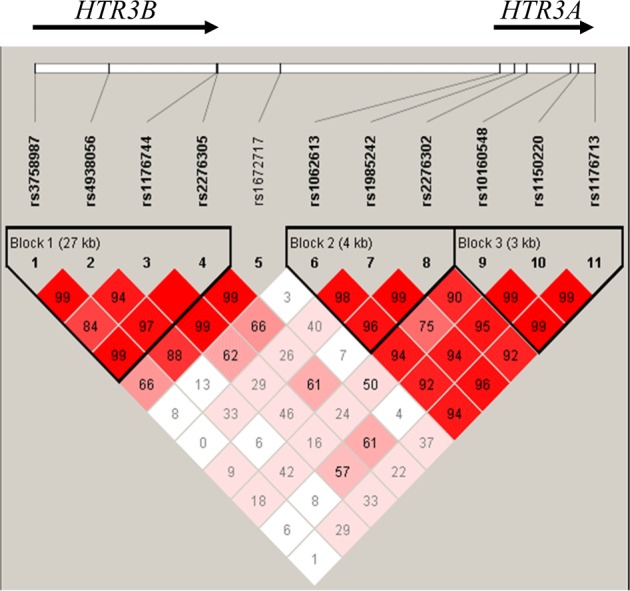
LD structure for *HTR3A* and *HTR3B* SNPs in Chinese Han smokers. The *D*′ values were calculated by Haploview (v. 4.2) shown in each box, and haplotype blocks were defined according to the criteria of Gabriel et al. (2002)^49^. The arrow at the top of the figure indicates the gene transcription direction from 5′ to 3′

On the basis of the definition of haplotype block by Gabriel et al.^49^, two blocks within *HTR3A* and one block within *HTR3B* were revealed (Fig. 1). Haplotype-based association analyses for all major haplotypes (frequency ≥ 5%) were performed in the three blocks mentioned above with FTND score. There was only one major haplotype, T-T-A-G, formed by SNPs rs3758987, rs4938056, rs1176744, and rs2276305, located in the 5′ region of *HTR3B*, with a frequency of 14.4%, which was significantly associated with FTND score under the additive model (Hap-score = 3.67; *p* = 0.00025) (Table 3). This association remained significant after Bonferroni correction for multiple testing. On the other hand, the other two major haplotypes in *HTR3A* did not show any significant association with the FTND score (*p* > 0.05).

**Table 3:** Major haplotypes (frequency > 0.05) within *HTR3B* and *HTR3A* associated with FTND in Chinese Han smokers

Gene	SNP combination	Haplotype	Hap-Freq	Hap-Score	*p*-Hap	*p*-Global
*HTR3B*	rs3758987-rs4938056-rs1176744-rs2276305	T-T-A-G	0.14	3.67	**0.00025**	**0.00547**
		C-T-C-G	0.14	−1.571	0.11535	
		T-T-A-A	0.26	−0.94	0.34542	
		T-C-A-G	0.40	−0.38	0.70192	
*HTR3A*	rs1062613-rs1985242-rs2276302	T-A-G	0.09	1.28	0.20068	0.52552
		C-A-A	0.18	−0.08	0.93544	
		C-T-A	0.71	−0.94	0.34864	
	rs10160548-rs1150220-rs1176713	G-A-G	0.10	1.35	0.17649	0.46057
		G-G-A	0.10	−1.28	0.1997	
		G-G-G	0.14	−0.16	0.87608	
		T-G-A	0.66	0.04	0.97098	

The results were adjusted for age, working time, and area under additive model. Significant association is shown in boldface type. The corrected *p* value after Bonferroni correction for four tested major haplotypes in *HTR3B* is 0.0125 (0.05/4)

### Gene–gene interaction analysis

As we know, *HTR3A*, *HTR3B*, and *SLC6A4* play critical roles in regulating serotonin signaling. Here we performed interaction analysis for 12 SNPs on the FTND score. As shown in Table 4, the best model displayed a significant genetic interaction effect with the FTND score. This significant interaction model included rs10160548 in *HTR3A*, and rs3758987, rs2276305, and rs1672717 in *HTR3B*, with a CVC of 8/10, prediction accuracy of 56.41%, and an empirical *p* value of 0.0074 based on 10^7^ permutation tests.

**Table 4:** Best SNP combination in *HTR3A* and *HTR3B* associated with FTND in Chinese Han smokers

SNP combination	ND measure	CVC	Prediction accuracy	Permutated *p* value
*HTR3A*: rs10160548 *HTR3B*: rs3758987, rs2276305, rs1672717	FTND	8/10	56.41%	0.0074

The empirical *p* value was calculated by 10^7^ permutations

*CVC* cross-validation consistency

### Associations among genotype, DNA methylation, and gene expression

**Fig. 2:**
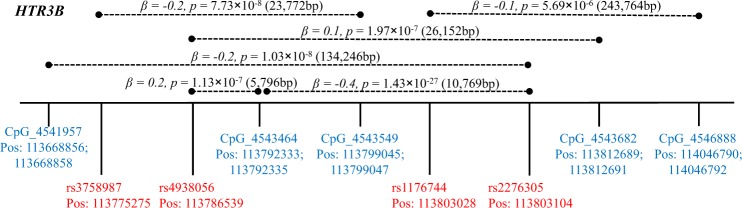
Schematic diagram of *cis-*mQTL analysis (within 50 kb upstream and downstream of each SNP) and position of investigated SNPs in *HTR3B*. Dashed lines refer to the significant SNP–CpG pairs. Pos genome position of SNP and CpG site

**Fig. 3:**
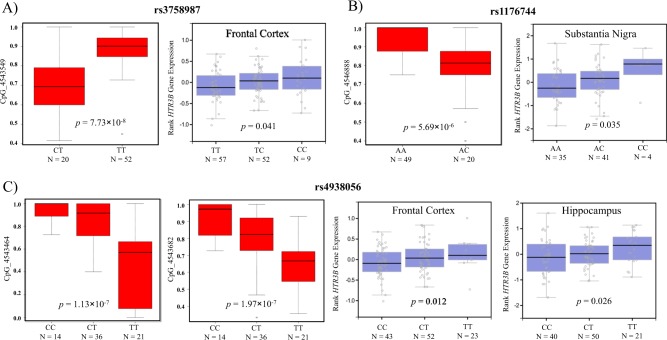
Association between variants and DNA methylation or *HTR3B* mRNA expression by *cis*-mQTL analysis or *cis*-eQTL analysis. **a** Cis-mQTL analysis between rs3758987 and CpG_4543549 and cis-eQTL between rs3758987 and HTR3B mRNA in frontal cortex. **b** Cis-mQTL analysis between rs1176744 and CpG_4546888 and cis-eQTL between rs1176744 and HTR3B mRNA in substantia nigra. **c** Cis-mQTL analysis between rs4938056 and CpG_4543464 or CpG_4543682 and cis-eQTL between rs4938056 and HTR3B mRNA in frontal cortex and hippocampus, respectively

The associations of the four SNPs in *HTR3B* with *cis*-mQTLs are shown in Supplementary Table 3. Figure 2 depicts the position of the SNPs and CpG sites. Of them, six pairs of significant SNP–CpG associations were detected, which were formed by four SNPs (rs2276305, rs3758987, rs4938056, and rs1176744) and five CpG sites (CpG_4543464, CpG_4541957, CpG_4543549, CpG_4543682, and CpG_4546888), with *p* values ranging from 1.43 × 10^−27^ to 5.69 × 10^−6^ (Fig. 3 and Supplementary Figure 2).

To confirm the results of *cis*-mQTL, a similar method was applied to perform *cis*-mQTL analysis by adjusting with the FTND score and age. The results were in accordance with the above found from the samples of both smokers and nonsmokers (Supplementary Table 2), which further demonstrated that these SNPs are risk variants affecting methylation in smokers.

Because the significant pairs of SNPs and CpG sites are located in *HTR3B* according to the *cis*-eQTL association dataset from GTEx Portal (https://gtexportal.org/home/), 12 brain regions were available to test eQTL. We found three (rs3758987, rs1176744, and rs4938056) of the four SNPs showed significant correlation with allele-specific mRNA expression of *HTR3B* in the human brain. The *p* values for the correlation of rs3758987 and *HTR3B* in the frontal cortex (Fig. 3A), of rs1176744 and *HTR3B* in the substantia nigra (Fig. 3B), of rs4938056, and of *HTR3B* in the frontal cortex and hippocampus (Fig. 3C) are 0.041, 0.035, 0.012, and 0.026, respectively.

## Discussion

In the present study, through a series of association analyses including individual SNP- and haplotype-based analyses, SNP-by-SNP interaction analysis, and *cis-*mQTLs analysis on ND among 12 SNPs within *HTR3A*, *HTR3B*, and *SLC6A4* in Chinese Han smokers, we found one SNP, rs1176744 in *HTR3B*, and one major haplotype, T-T-A-G, formed by rs3758987, rs4938056, rs1176744, and rs2276305 in *HTR3B*, to be significantly associated with the FTND score under the additive genetic model. The SNP-by-SNP interaction analysis showed that there existed significant interaction in affecting ND among SNPs rs10160548 in *HTR3A*, and rs3758987, rs2276305, and rs1672717 in *HTR3B*. We also identified four methylation sites (i.e., CpG_4543549, CpG_4543464, CpG_4543682, and CpG_4546888) associated significantly with three expression and methylation quantitative trait loci (*cis*-meQTL) SNPs (rs3758987, rs4938056, and rs1176744), which are located within the major haplotype detected in *HTR3B*.

Given that there exist various genetic architectures and smoking behaviors underlying ND in different ethnic populations^55^ and a high smoking prevalence in Chinese men^8^, it is essential to discover the pathogenic variants of ND in the Chinese Han population. To our knowledge, this is the first study reporting the presence of significant associations of serotonergic genes with ND in Chinese Han populations. These findings highlight an important role of the variations in serotonin transporter and receptors, which govern the *trans*-synaptic serotonergic signaling underlying the pathophysiology of ND.

Rs1176744 is a known functional variant (Tyr129Ser) in *HTR3B*, which results in a change of tyrosine to serine at the 129th amino-acid residue of 5-HT_3B_^56^. Here we found that the rs1176744 was the only SNP showing significant association with ND at the individual level. Together with rs3758987, rs4938056, and rs2276305, they consist of a major haplotype, T-T-A-G, which is associated significantly with ND. We also found that this SNP acts as a *cis*-mQTL to alter the extent of methylation of CpG_4546888 in blood and *cis*-eQTL to modulate the mRNA expression of *HTR3B* in the substantia nigra. However, no significant interactive effects of rs1176744 with other SNPs were detected. Previously, although we showed that there exists no significant association of rs1176744 with ND in European-American (EA), African-American (AA), and pooled samples, we did find significant interaction of this SNP with others in *HTR3A* and *SLC6A4*, indicating an important role of these genes and variants in ND^32^. Such inconsistent results might be attributable to the difference in allele frequencies across different populations. For instance, the MAF of rs1176744 is 0.48 for EA, 0.33 for AA, and 0.17 for the Chinese Han population. The inconsistent MAF among different ethnic samples is rather common, which may reflect a different evolution rate and biological importance. In addition, another study of ours showed not only that SNP rs1176744 along with rs11214769 in *HTR3B* formed a major haplotype that is significantly associated with ND in the EA sample but also a significant interactive effect among rs1176744 in *HTR3B*, rs897685 and rs7118530 in *HTR3A*, and rs25528 in *SLC6A4* on ND in the AA sample^36^. Further, rs1176744 proved to be significantly associated with alcohol dependence in the AA sample^33^. For the abovementioned results, the most reasonable explanation is the presence of heterogenity in these ethnic populations, and we for the first time show that SNP rs1176744 is active in ND in the Chinese Han population. Based on the power analysis results, we have 98.48% power to detect a polymorphism of rs1176744 (with a MAF of 0.17) with the sample size of 2616 used in this study. Although the individual SNP association analysis yielded a nominal significance of rs1176744, we had 98.48% power to support the lack of significance.

Haplotype-based association analysis revealed that one major haplotype, T-T-A-G, constituted by rs3758987, rs4938056, rs1176744, and rs2276305 in *HTR3B*, was significantly associated with the FTND score (*p* = 0.00025). Moreover, our data showed that all SNPs included in this haplotype can regulate methylation of nearby CpG sites (CpG_4543549, CpG_4543464, CpG_4543682, and CpG_4546888). The significant association of SNPs in one major haplotype with *cis*-mQTL indicated that the major haplotype, T-T-A-G, in *HTR3B* plays an important role in determining the pathology of ND through influencing the methylation of adjacent CpG sites in Chinese male smokers.

The three serotonergic genes, i.e., *HTR3A*, *HTR3B*, and *SLC6A4*, have similar functions or are known to interact^57^. Thus, those with no obviously associated polymorphisms might exert genetic effects on ND when the epistatic effects are taken into consideration. In this study, by applying our own developed GMDR-GPU program^51^, we discovered that the best variant combination, consisting of four SNPs (rs10160548 in *HTR3A*, and rs3758987, rs2276305, rs1672717 in *HTR3B*), showed significant epistatic effects on ND (*p* = 0.0074), although the SNPs presented no important associations with ND. Previously, we showed that rs10160548 was significantly associated with SQ and heaviness of smoking index in an AA sample, and there existed significant interactive effects of this SNP with others on ND^32^. Furthermore, our group showed that the variants within the three genes exerted significant epistatic effects with several highly related ND assessments in EAs, AAs, and the pooled samples^32^. This suggests that these serotonergic genes are likely producing their effects on ND by epistasis, which might well explain why they could not be detected by association analysis.

**Fig. 4:**
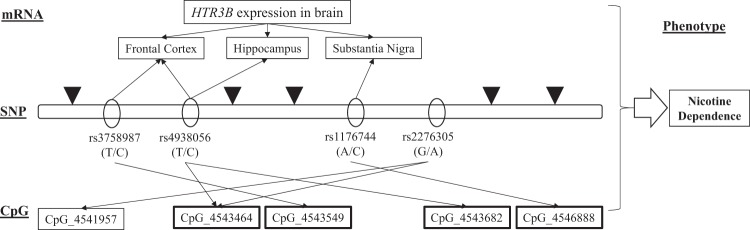
Overall schematic diagram of SNPs in *HTR3B*, which regulates methylation loci and mRNA expression associated with smoking. Four SNPs formed as a haplotype in *HTR3B* significantly associated with five CpG sites. Allele-specific mRNA expression of *HTR3B* in the ND-related brain regions were also found, which will affect ND in Chinese Han smoker

Cigarette smoking as an exogenous modifier can change DNA methylation^58^, and genetic variants at specific loci can influence both regional and distant DNA methylation^59^. By linking genetic variants, DNA methylation, mRNA expression, and ND, we can explore the possible biological mechanisms of ND. By using a dataset with all *CHRNA5* methylation, expression, and risk of SNPs data available, Hancock et al. first revealed that several genetic variations underlying *CHRNA5* methylation in the human brain contributed to the risk of ND^60^. Recently, we linked SNP, *cis*-mQTL, and *cis*-eQTL^45^ and revealed two methylated sites associated significantly with three SNPs forming four significant CpG–SNP pairs in a Chinese Han population. In the present study, as shown in Fig. 4, five methylated sites associated with four SNPs in one haplotype were identified. Further, based on GTEx Portal (https://gtexportal.org/home/), a database that provides information on human gene expression and regulation and its relation to genetic variation, we identified three *cis*-meQTLs that affected both the extent of methylation and mRNA expression of *HTR3B*. Rs3758987 and rs4938056 were significantly correlated with *HTR3B* mRNA expression in the human frontal cortex (*p* = 0.041 and 0.012, respectively). The frontal cortex is involved in ND, which is strongly associated with neurotransmitter release^61^. We also found that rs4938056 and rs1176744 presented significant relativity with mRNA expression of *HTR3B* in the hippocampus and substantia nigra, respectively. These two brain regions are known to be related to ND^62–64^. Thus, these SNPs may be risk targets regulating the expression of *HTR3B* by altering the methylation status.

To the best of our knowledge, this is the first study to demonstrate that variants in serotonergic genes are significantly associated with ND in the Chinese Han population. From individual SNP, haplotype, and gene–gene interactions, and by linking DNA methylation and mRNA expression to perform *cis*-mQTL and *cis*-eQTL analyses, we explored the possible biological pathology involved in ND. Our results indicate that the individual SNP rs1176744 (Tyr129Ser) was nominally significant and a major haplotype formed by rs1176744, rs3758987, and rs4938056 in HTR3B showed significant association with the FTND score. Further, four methylation sites were found to be significantly associated with three *cis*-meQTL SNPs. These findings extend our knowledge of the genetic roles of serotonin in affecting ND, which will help researchers look for targets that account for ND in Chinese smokers.

## Electronic supplementary material

Supplementary Tables

Supplementary Figures

Supplementary Figures Legends
