# Supplementary material for: Association and cis-mQTL analysis of variants in serotonergic genes associated with nicotine dependence in Chinese Han smokers
Source: Transl Psychiatry. 2018 Nov 7;8:243. doi: 10.1038/s41398-018-0290-8 (PMC6221882; doi:10.1038/s41398-018-0290-8)
Supplement: Supplementary file 1 — Supplementary Tables [file 41398_2018_290_MOESM1_ESM.docx]

**Table S1**. The distribution of FTND score with smokers

| Characteristic of FTND | | |
| --- | --- | --- |
|  | FTND Score | Sample Size |
| Light smoker  (N = 1,373) | 0 | 1 |
|  | 1 | 1 |
|  | 2 | 8 |
|  | 3 | 241 |
|  | 4 | 337 |
|  | 5 | 785 |
| Heavy smoker  (N = 1,243) | 6 | 751 |
|  | 7 | 290 |
|  | 8 | 130 |
|  | 9 | 62 |
|  | 10 | 10 |

**Table S2.** Detailed information for each selected SNP in *HTR3A*, *HTR3B* and *SLC6A4*

| Chromosome | Position | Gene Symbol | SNP ID | SNP Type | Alleles (Major/minor) | MAF | References |
| --- | --- | --- | --- | --- | --- | --- | --- |
| 11 | 113845541 | *HTR3A* | rs1150226 | Intron | G/A | 0.12 | [^1-3^](#_ENREF_1) |
|  | 113846006 |  | rs1062613 | 5'UTR | C/T | 0.09 |  |
|  | 113846077 |  | rs33940208 | Synonymous | C/T | 0.16 |  |
|  | 113848273 |  | rs1985242 | 5'UTR | T/A | 0.28 |  |
|  | 113850140 |  | rs2276302 | Intron | A/G | 0.10 |  |
|  | 113856681 |  | rs10160548 | Intron | T/G | 0.34 |  |
|  | 113857886 |  | rs1150220 | Intron | G/A | 0.09 |  |
|  | 113860425 |  | rs1176713 | Synonymous | A/G | 0.24 |  |
|  | 113775275 | *HTR3B* | rs3758987 | Intron | T/C | 0.17 |  |
|  | 113780981 |  | rs11606194 | Intron | T/C | 0.04 |  |
|  | 113786539 |  | rs4938056 | Intron | T/C | 0.41 |  |
|  | 113803028 |  | rs1176744 | Missense | A/C | 0.17 |  |
|  | 113803104 |  | rs2276305 | Synonymous | G/A | 0.27 |  |
|  | 113807607 |  | rs3782025 | Intron | A/G | 0.36 |  |
|  | 113812733 |  | rs1672717 | Intron | A/G | 0.38 |  |
|  | 113816377 |  | rs17614942 | Intron | C/A | 0.05 |  |
| 17 | 28525011 | *SLC6A4* | rs1042173 | 5'UTR | C/A | 0.20 |  |

**Table S3**. SNPs in *HTR3B* associated with methylation level at nearby CpGs (±25kb) in blood of Chinese Han population

| SNP | SNP’s position | CpG | CpGs position | | Distance to SNP (bp) | All selected samples (N=72) | | Smokers (N=36) | |
| --- | --- | --- | --- | --- | --- | --- | --- | --- | --- |
|  |  |  | Start | End |  | Beta | *p* value | Beta | *p* value |
| rs2276305 | 113803104 | CpG_4543464 | 113792333 | 113792335 | -10769 | -0.4 | 1.43E-27 | -0.4 | 2.04E-14 |
|  |  | CpG_ 4541957 | 113668856 | 113668858 | -134246 | -0.2 | 1.03E-08 | -0.2 | 4.34E-03 |
| rs3758987 | 113775275 | CpG_4543549 | 113799045 | 113799047 | 23772 | -0.2 | 7.73E-08 | -0.1 | 2.31E-02 |
| rs4938056 | 113786539 | CpG_4543464 | 113792333 | 113792335 | 5796 | 0.2 | 1.13E-07 | 0.3 | 3.39E-05 |
|  |  | CpG_4543682 | 113812689 | 113812691 | 26152 | 0.1 | 1.97E-07 | 0.2 | 5.91E-04 |
| rs1176744 | 113803028 | CpG_4546888 | 114046790 | 114046792 | 243764 | -0.1 | 5.69E-06 | -0.2 | 3.62E-04 |

Note: Distance to SNP means the distance from associated SNP to CpG site. Age and FTND score were used as covariates to adjust the methylation level for all samples, and age as covariate to adjust the methylation level for smokers to validate the CpGs found in all samples.

**References**

1. Yang J, Li MD. Association and interaction analyses of 5-HT3 receptor and serotonin transporter genes with alcohol, cocaine, and nicotine dependence using the SAGE data. *Hum Genet* 2014; **133**(7)**:** 905-918.

2. Seneviratne C, Franklin J, Beckett K, Ma JZ, Ait-Daoud N, Payne TJ *et al.* Association, interaction, and replication analysis of genes encoding serotonin transporter and 5-HT3 receptor subunits A and B in alcohol dependence. *Hum Genet* 2013; **132**(10)**:** 1165-1176.

3. Yang Z, Seneviratne C, Wang S, Ma JZ, Payne TJ, Wang J *et al.* Serotonin transporter and receptor genes significantly impact nicotine dependence through genetic interactions in both European American and African American smokers. *Drug Alcohol Depend* 2013; **129**(3)**:** 217-225.
