# Supplementary figures and images for: Association and cis-mQTL analysis of variants in serotonergic genes associated with nicotine dependence in Chinese Han smokers
Source: Transl Psychiatry. 2018 Nov 7;8:243. doi: 10.1038/s41398-018-0290-8 (PMC6221882; doi:10.1038/s41398-018-0290-8)

## Slide 1
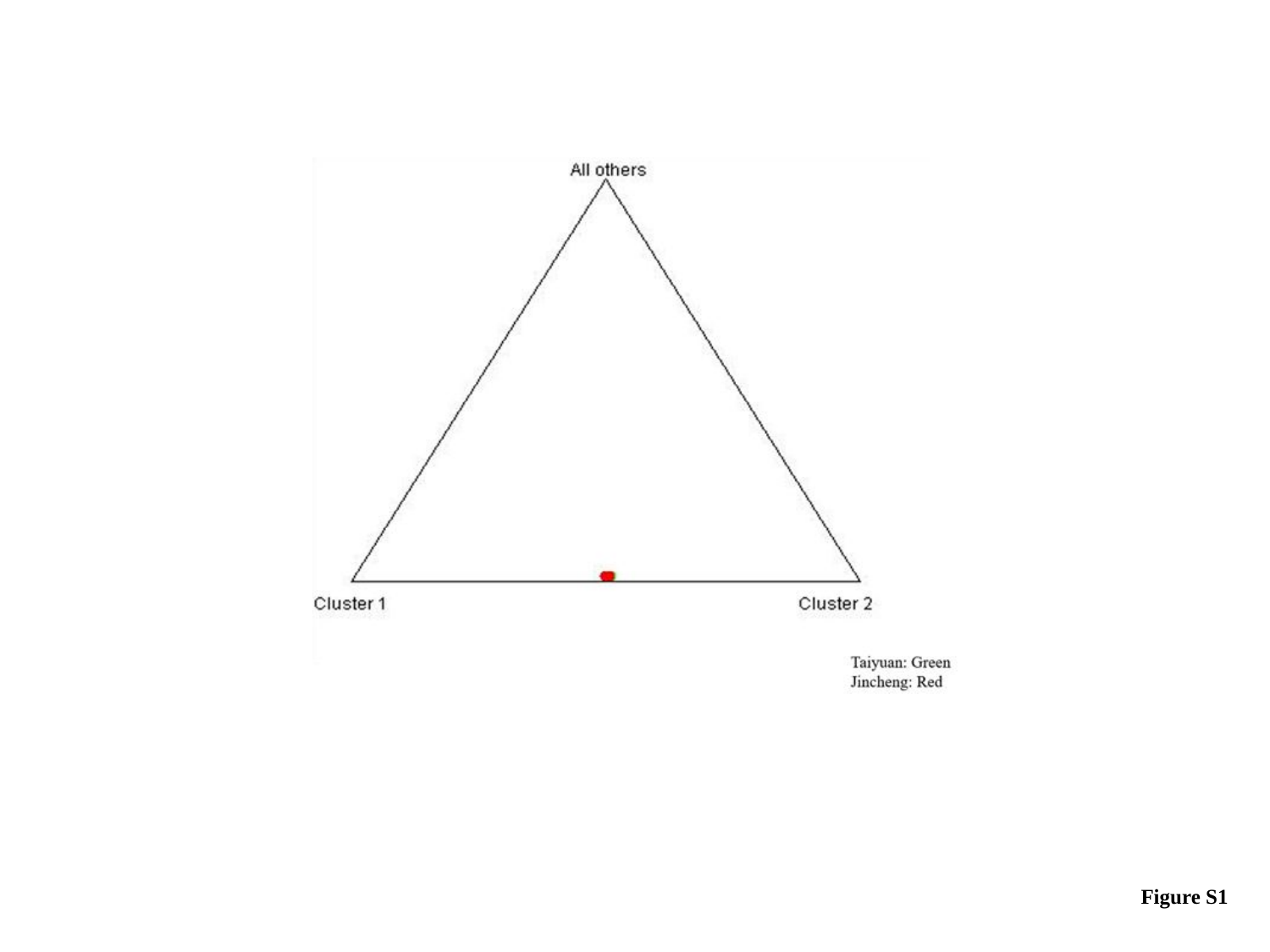

Figure S1

## Slide 2
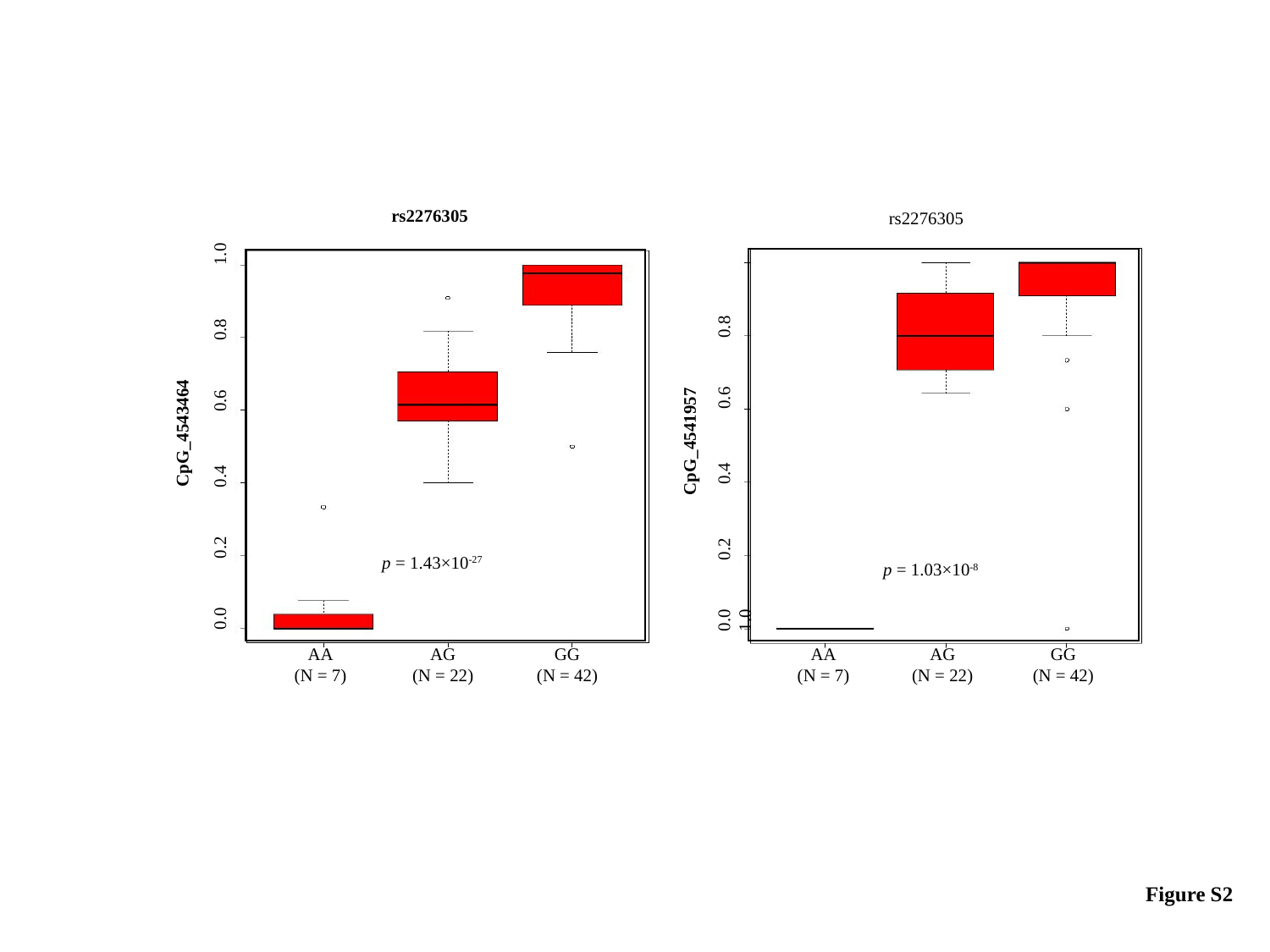

0.0 0.2 0.4 0.6 0.8 1.0
rs2276305
CpG_4543464
p = 1.43×10-27
AA
(N = 7)
AG
(N = 22)
GG
(N = 42)
rs2276305
0.0 0.2 0.4 0.6 0.8 1.0
CpG_4541957
p = 1.03×10-8
AA
(N = 7)
AG
(N = 22)
GG
(N = 42)
Figure S2

Supplement: Supplementary file 2 — Supplementary Figures [file 41398_2018_290_MOESM2_ESM.pptx]
