# Supplementary material for: Association and cis-mQTL analysis of variants in serotonergic genes associated with nicotine dependence in Chinese Han smokers
Source: Transl Psychiatry. 2018 Nov 7;8:243. doi: 10.1038/s41398-018-0290-8 (PMC6221882; doi:10.1038/s41398-018-0290-8)
Supplement: Supplementary file 3 — Supplementary Figures Legends [file 41398_2018_290_MOESM3_ESM.docx]

**Legends to Supplementary Figures**

**Figure S1.** Clustering results based on 30 ancestry-informative markers of Chinese Han population. Red represents the samples from Taiyuan and green the samples from Jincheng.

**Figure S2.** The *cis*-mQTL analysis between rs2276305 and extent of methylation in blood of 72 Chinese Han participants.
